# Reforming Food, Drug, and Nutraceutical Regulations to Improve Public Health and Reduce Healthcare Costs

**DOI:** 10.3390/foods14132328

**Published:** 2025-06-30

**Authors:** Sunil J. Wimalawansa

**Affiliations:** CardioMetabolic & Endocrine Institute, North Brunswick, NJ 08902, USA; suniljw@hotmail.com

**Keywords:** artificial intelligence, health insurance, healthcare cost, hospital sector, innovations, nutrients, nutrition, public health

## Abstract

Neglecting preventive healthcare policies has contributed to the global surge in chronic diseases, increased hospitalizations, declining quality of care, and escalating costs. Non-communicable diseases (NCDs)—notably cardiovascular conditions, diabetes, and cancer—consume over 80% of healthcare expenditure and account for more than 60% of global deaths, which are projected to exceed 75% by 2030. Poor diets, sedentary lifestyles, regulatory loopholes, and underfunded public health initiatives are driving this crisis. Compounding the issue are flawed policies, congressional lobbying, and conflicts of interest that prioritize costly, hospital-based, symptom-driven care over identifying and treating to eliminate root causes and disease prevention. Regulatory agencies are failing to deliver their intended functions. For instance, the U.S. Food and Drug Administration’s (FDA) broad oversight across drugs, devices, food, and supplements has resulted in inefficiencies, reduced transparency, and public safety risks. This broad mandate has allowed the release of unsafe drugs, food additives, and supplements, contributing to the rising childhood diseases, the burden of chronic illness, and over-medicalization. The author proposes separating oversight responsibilities: transferring authority over food, supplements, and OTC products to a new Food and Nutraceutical Agency (FNA), allowing the FDA to be restructured as the Drug and Device Agency (DDA), to refocus on pharmaceuticals and medical devices. While complete reform requires Congressional action, interim policy shifts are urgently needed to improve public health. Broader structural changes—including overhauling the Affordable Care Act, eliminating waste and fraud, redesigning regulatory and insurance systems, and eliminating intermediaries are essential to reducing costs, improving care, and transforming national and global health outcomes. The information provided herein can serve as a White Paper to help reform health agencies and healthcare systems for greater efficiency and lower costs in the USA and globally.

## 1. Introduction

Healthcare costs are rising globally, with a rapid increase in Western countries, particularly in the USA, due to reliance on a “sick-care” model that prioritizes treatment over prevention. This ineffective approach escalates costs without improving care quality or outcomes. Instead, the healthcare model should transition towards “disease prevention” and the maintenance of public health. Such an approach will reduce hospitalizations [1,2]—the primary driver of healthcare expenses (see Section 4.3), while decreasing school and workplace absenteeism and boosting productivity. Achieving this requires systemic structural changes, which includes public education on nutrition, healthy lifestyles and minimizing harmful habits, ensuring access to affordable nutritional and functional nutraceuticals and healthy food across the country, major restructuring of the Food and Drug Administration (FDA), and new legislation to support preventive healthcare initiatives and redistribution of funding.

Unhealthy lifestyles, such as poor diets, sedentary lifestyles, and high-risk activities [3,4], and the lack of public health measures to prevent diseases keep society sick, increasing hospitalizations and escalating healthcare costs [5,6]. Implementing cost-effective, transformative measures in food, behavior, and public health, alongside effective oversight, could yield significant clinical improvements and tangible benefits in the population’s health and reduce healthcare costs and burdens. Taking these steps is somewhat analogous to introducing an effective vaccine, or a targeted micro-nutrient supplement (or food fortification) n specific regions, leading to modifying clinical outcomes already practiced in some countries.

More importantly, these systemic initiatives would reduce the incidence of chronic disease, thereby reducing healthcare spending by an estimated one-third within four years, saving an estimated one trillion dollars in healthcare costs. With federal and state governments’ support, a critical opportunity exists to enhance national health (physical and mental health), the economy—reduce absenteeism and enhance productivity. Achieving this requires steadfast commitment, long-term vision, and systemic reform [7].

Shifting from a reactive, sickness-driven healthcare model to a proactive, prevention-focused approach would position the US as a global leader in health reform [8], delivering better outcomes at lower costs while ensuring a healthier, more sustainable future—a complete paradigm shift. Such a transition from a reactive, sickness-care approach to a proactive, disease-prevention-oriented healthcare model would position the US as a global leader in health reform [8,9], providing better clinical outcomes at less cost. That would promise healthier populations, reduced costs, increased productivity, and a sustainable future for generations. Innovations in health care should incorporate artificial intelligence (AI) and machine learning (ML), and cybernetics, and be translatable from the current reactive interventions to proactive disease prevention [8]. This would maximize returns on investment with the most benefit to the population and to the country [9].

### 1.1. The Root Causes That Escalate Healthcare Costs

This review discusses several key causes that drive escalating healthcare costs. Paradoxically, no industry or entity, including government agencies, currently focuses on identifying and eliminating the root causes of chronic diseases [10]. Virtually all companies direct their research and development toward profitable treatments that alleviate symptoms, diagnostic methods, and life-prolonging interventions—the lucrative path [3] not eradicating diseases or promoting the health span. In addition, malnutrition aggravates chronic diseases and impairs the ability to recover from infections and non-communicable diseases [11]. As a result, critical factors, such as poor nutrition, particularly micro-nutrient deficiencies, as well as imbalance of other essential nutrients, food additives, incorporation of environmental toxins (like aluminum, microplastic, and glyphosate), chronic inflammation [12], and lifestyle modifications are largely overlooked [3,11].

Proper nutrition with a balanced diet can mitigate various disorders that arise from food and environmental contaminants [11,13]. Examples of such disorders include autism spectrum disorder, autoimmune disorders, chronic brain diseases, cancers, asthma, cardiometabolic disorders, and reproductive ill health [13]. In addition, dietary guidelines are conflicting and outdated [14,15,16,17,18,19]. Almost current nutrition guidelines are outdated. Recommendations rely on universal references that follow a “one-size-fits-all” model, which should not be the case. Guideline committees often adopt these standardized approaches under the influence of pharmaceutical companies (discussed later) and evidence-based medicine (EBM). These shortcut methods fail to address the diverse needs of different communities and countries [17].

For example, recommendations for micro-nutrients like vitamin D should be made individually on a person’s body size (BMI or body weight) and age, not arbitrarily using one dose for all, as is the case today [20,21]. Besides, the narrow emphasis/focus on symptom management rather than addressing underlying causes leads to excessive medicalization and a lifelong cycle of chronic disease management, costly—prioritizing treatment over prevention, reversal, and eradication [11,12,22,23]. These flawed methods, aimed at prolonging life rather than preserving health, are estimated to increase healthcare costs nearly tenfold. Instead, clinical guidelines should not use EBM that are designed to prioritize medications (not health), be devoid of any pharmaceutical industry bias, and be patient-centric, individualized based on body size and lifestyle [3], as are the concepts in integrative medicine [24].

### 1.2. The Role of Nutrition in Health and Disease

Dietary interventions have emerged as promising adjunct strategies for managing metabolic disorders, autoimmune diseases, cardiovascular diseases, cancer, and neurodegenerative conditions [14,15,18,19,25], as well as reducing all-cause mortality [25]. A proper and balanced diet can influence key metabolic pathways, slow disease progression, enhance therapeutic responses, and even reverse disease courses [25].

Nutrient interventions can also modulate metabolic reprogramming (aiding regenerative medicine) and, thus, play a crucial role in slowing (or reversing) cancer progression. Dietary modifications can also positively impact tumor metabolism, local invasion, metastasis, and as an adjunct therapy efficacy [14,15,26]. However, the complex relationship between diet and disease pathogenesis remains poorly understood, leading to the underutilization of diet and orthomolecular-based therapies [19]. Strategies such as calorie restriction, prolonged overnight fasting, fasting-mimicking diets, specific amino acid therapies, ketogenic diets, and macronutrient modifications have demonstrated immunomodulatory and molecular benefits, improving disease management with little cost [19,25,26].

### 1.3. Preventing Chronic Diseases via Proper Nutrition

Neglecting disease prevention in global healthcare policies has promoted rising illness [6,27], hospitalizations, morbidities, opportunity costs, and premature deaths, driving up costs while diminishing care quality due to the adherence to the sick-care model [28]. Despite strong evidence supporting that prevention is significantly better [27], efforts to address root causes—the underlying reasons for the surge in chronic diseases—remain significantly inadequate [29]. Public health initiatives, which could significantly ease healthcare burdens, are often sidelined and overlooked [3] as the pharmaceutical industry lobby steers policymakers and healthcare professionals toward treatment-focused rather than prevention-based approaches [30,31].

It is well known that individual behaviors contribute to health promotion and/or disease. Adopting and maintaining healthful behaviors, such as good eating habits and regular physical exercise, relaxation, and modifying harmful behaviors like illegal drugs, smoking, and alcohol, significantly reduces the risks of most chronic diseases [27]. In addition, the disease-preventative approach encompasses adopting safe, effective, and affordable lifestyles, taking appropriate supplements, like vitamin D, omega-3 fatty acids, essential minerals, etc., and other essential micro-nutrients [32,33], a balanced diet, daily physical activities, stress management, and integrative therapies [24]. Collectively, these promote health and prevent diseases.

### 1.4. Enhancing Health Through Improved Food Regulation

Food and nutraceuticals are crucial in health promotion and disease prevention, yet regulatory oversight remains inconsistent. Nutraceuticals, which include functional foods, dietary supplements, and bioactive compounds, offer potential benefits in managing chronic diseases, enhancing immune function, and supporting overall well-being. However, the lack of standardized quality control, misleading marketing claims, and insufficient consumer education hinder their optimal use [34]. The lack of updated laws related to food and nutrients and inconsistent enforcement by the Food and Drug Administration (FDA) make them worse. Strengthening data-based regulations and ensuring transparency in labeling and efficacy claims are essential to maximizing their benefits while preventing misinformation and exploitation.

The FDA’s current approach to food and functional nutraceutical oversight is fragmented and inconsistent, often prioritizing pharmaceuticals while neglecting preventive processes, especially nutrition. A streamlined regulatory framework emphasizing scientific validation, safety monitoring, and clear labeling would empower consumers and healthcare professionals to make informed choices. Additionally, integrating nutraceutical and orthomolecular approaches into mainstream healthcare as an adjunctive therapy could significantly reduce healthcare costs by shifting the focus from treatment to prevention. Reforming policies to eliminate conflicts of interest [35] and ensuring unbiased research funding will be key steps in optimizing their role in public health.

### 1.5. Integrating Technology, Artificial Intelligence, and Cybernetics into Healthcare

Governments worldwide often underinvest in disease prevention despite the growing needs of aging populations whose immune defenses naturally decline over time, making them more susceptible to sicknesses. Relying predominantly on reactive, hospital-centric healthcare models has proven economically unsustainable [36] and insufficient for ensuring long-term population health [37,38].

For example, healthcare providers should use AI to triage, provide rapid availability of accurate medical records, streamline patient administration, and support expedited clinical decision-making processes. Today, with appropriate input, AI programs can generate highly personalized diets—more accurately and efficiently than trained healthcare professionals—not only for healthy individuals but, more importantly, for those with specific diseases. However, any AI must be applied with common sense, ensuring it complements patient monitoring, disaster management, cybernetics, and healthcare interventions and innovations.

AI should serve to manage the health system in a central role and function as an enabling tool, not necessarily a decision-maker in clinical management [38]. Currently, AI is primarily used to streamline operations and optimize revenue and administrative management. However, both AI and ML have vast potential across many other facets of healthcare [38], including diagnosis, treatment planning, patient monitoring, and personalized medicine. For more complicated tasks, such as robotics, deliveries, and supply chains in the health system, AI augmentation should be used [39]. This article discusses how this system can be developed based on a realistic assessment of current AI technologies, with a future vision, predicted developments, and expected clinical outcomes.

A transformative shift is imperative—one that emphasizes preventive care and leverages technological advancements to enhance healthcare delivery. AI stands at the forefront of this transformation, offering tools for rapid diagnosis [39], predictive analytics [37], and efficient resource allocation. AI applications can reduce unnecessary and over-testing, improve diagnostic accuracy [38], prevent prescription and medical errors [38], enhance emergency and operating room efficiency, and streamline clinical workflows [37,38]. For instance, AI-driven digital pathology enables faster and more precise disease detection and rapid diagnosis, which are crucial for timely interventions [37].

Beyond diagnostics, integrating AI and automation across agencies, hospitals, and governmental and private sectors can address systemic inefficiencies, including identifying and rapidly eliminating duplications, fraud and waste [38]. Such integration facilitates real-time data analysis, enhances transparency, and supports proactive decision-making, strengthening overall healthcare infrastructure [40]. The COVID-19 pandemic underscored the urgent need for such advancements [39]. AI technologies were pivotal in early outbreak detection, contact tracing, rapid diagnosis, and resource management, highlighting their role in enhancing public health responses [40].

Policymakers must implement major reforms to reduce healthcare costs and prioritize preventive strategies to achieve meaningful savings and long-term health benefits. This includes implementing nutritional education programs in schools, colleges, and in the community [38], ensuring access to wholesome foods, eliminating harmful foods and additives, promoting regular physical activity, and facilitating routine, low-cost health screenings [36]. Evidence suggests that such measures can significantly reduce the incidence of chronic diseases and associated healthcare costs [36].

Investing in public health infrastructure and community-based prevention programs empowers individuals to maintain better health throughout their lifespan. By shifting the focus from reactive treatment to proactive prevention, governments can enhance national productivity, reduce healthcare expenditures, and build a more resilient healthcare system capable of withstanding future challenges.

## 2. Food and Nutraceuticals for Health and Disease Prevention

A well-balanced diet rich in essential nutrients is fundamental to maintaining good health and preventing chronic diseases [41]. Diets containing nutrient-dense food sources—whole foods, including fruits, vegetables, lean proteins, and healthy fats—provide vital macro-nutrients and micro-nutrients that support immune function, metabolic balance, and cellular repair [41]. Deficiency or excessive consumption of both micro- and macro-nutrients can lead to adverse health outcomes and, thus, should be avoided [42]. Nutraceuticals from natural food sources offer additional bioactive compounds that can enhance physiological functions, reduce inflammation, and mitigate the risk of cardiovascular disease, diabetes, and neurodegenerative disorders. Incorporating nutrient-dense foods and targeted nutraceuticals into daily diets can help individuals optimize health and reduce the burden of lifestyle-related diseases [41,43].

Beyond basic nutrition, specific nutraceuticals such as omega-3 fatty acids, hydrolyzed proteins, antioxidants (polyphenols), carotenoids (lycopene), prebiotics and probiotics, and adaptogens have demonstrated promising effects in modulating immune responses, improving gut health, and enhancing cognitive functions [34]. Emerging research highlights their potential, even to reverse disease progression by influencing key metabolic pathways and reducing oxidative stress [41]. Personalized nutrition approaches that integrate functional foods and nutraceuticals can improve health outcomes by addressing nutritional deficiencies and genetic predispositions [41,43]. Individuals can proactively manage their health and enhance overall well-being by prioritizing food as medicine and leveraging the benefits of nutraceuticals [34,42].

### 2.1. Reevaluating the Safety of Preservatives and Food Additives

People often assume that food additives and preservatives approved by the US Food and Drug Administration (FDA) under “The Federal Food, Drug, and Cosmetic Act” (FDCA) are safe, but this is not necessarily the case [44]. The Delaney clause of the FDCA states that the FDA may not approve any food additive—even one deemed *safe* by the FDA—if it is “found to induce cancer” in laboratory animals [45]. Consequently, the Delaney Clause in the FDCA allows consumer activists to force the FDA to remove certain additives based on their purportedly carcinogenic activities [46]. Under the same Delaney Clause, in January 2025, the FDA banned Red Dye No. 3 from use in food, beverages, and certain drugs [47]. Red Dye No. 3 (a synthetic dye, erythrosine, derived from petroleum), which is a step toward eliminating toxic additives, including harmful preservatives introduced by the food industry [47].

Epidemiological studies have linked Red Dye No. 3 to adverse effects such as hyperactivity and neurobehavioral changes in children [47]. Animal research suggests it may also contribute to cancers, such as thyroid cancer [47]. Several other additives also pose significant health risks, including titanium dioxide, brominated vegetable oil, propylparaben, and potassium bromate. Ironically, these substances are banned in the European Union (EU) but remain approved by the FDA in the USA [48]. The state of West Virginia is the first to ban these harmful additives in the nation [49].

At high doses, titanium dioxide (TiO_2_; E171), a food additive used as a preservative, causes inflammation, exacerbates inflammatory bowel syndrome, and the formation of colon tumors [50,51]. Titanium, a white pigment, is one of the brightest pigments. It is used to making foods more vibrant and bright, and to prevent UV degradation [52]. However, the FDA does not mandate food makers to use the chemical name on ingredients in the additive list [52]. Instead of naming the additive, it might say color added, artificial colors, artificial color added, etc.—another loophole that must be rectified.

Parabens are another family of chemicals (methylparaben, propylparaben, butylparaben, and ethyl-paraben), frequently used as preservatives in cosmetic products to prevent the growth of bacteria and mold [53]. They are added to many skincare and haircare products as well as to some processed foods.

Notably, there are no special rules that the FDA applies to cosmetic preservatives. The rules treat preservatives in cosmetics like other cosmetic ingredients [53]. The public has expressed concersd about the harmful effects of preservatives and food additives. Through an FDA rule (a loophole) generally recognized as safe (GRAS), food companies continue adding new chemicals and additives without the FDA’s oversight or approval [54]. For example, animal studies have reported that parabens disrupt hormonal actions and reduce fertility; they may cause breast cancer risks [55,56]. Parabens are also used as an antimicrobial, a flavoring and a preservative agents used in foods [53].

### 2.2. Other Food Additives—FDA Responsibilities to Keep the Nation Safe

For the greater good and reassurance of the public, the proposed new entity, the new Food and Nutraceutical Agency (FNA) (see Section 7) must take a fresh look to reevaluate all preservatives and additives. In doing so, it would be responsible for eliminating and banning those proven to have harmful effects, which the FDA failed to do, particularly those agents banned in other countries [54,57]. For example, bleached flour (e.g., using chlorine gas or benzoyl peroxide), propylparaben, dough conditioners (potassium bromate and azodicarbonamide), brominated vegetable oil, butylated hydroxyanisole (BHA) and butylated hydroxytoluene (BHT) added to cereal, synthetic food dyes, such as blue 2, yellow 5, and red 40, antibiotics, cholesterol-free fat substitutes (olestra or Olean), synthetic hormones (like rBGH and rBST), as well as PFAS or per- and poly-fluoroalkyl substances (synthetic chemicals with grease, water, and heat-resistant properties). PFAS are widely used in fast-food wrappers, microwave popcorn bags, and takeout containers [54]. These chemicals are banned in many countries, but they are allowed to be used freely in the USA [57].

Potassium bromates (potassium bromate-propyl-paraben; KBrO_3_) is an oxidizing agent frequently used as a food additive. Animal studies have reported that it is a potential carcinogen for renal and testicular tissues [58,59]. In Europe, Canada, and China, KBrO_3_ is banned, but not in the USA. In the USA, it is used as an oxidizing agent to help mature flour (brominated flour) grow faster via chemical processes [59]. Besides, potassium bromate is used in the bakery industry to bind gluten molecules, increase elasticity, provide texture to the bread, and maintain shape [58,59]. There are more examples of food additives banned in the European Union but not in the USA. Titanium dioxide, artificial dyes, aspartame, azodicarbonamide (ADA), propyl gallate, sodium nitrite, methylene chloride, trichloroethylene, and ethylene dichloride— have all been shown to be harmful to humans [57]. The procrastination of the FDA to take action in this regard is puzzling.

Bromine, oil-brominated vegetable oil (BVO), is also added to vegetables, which can accumulate in substantial quantities in fatty tissues and has been reported to harm the nervous system [60]. BVO has also been shown to worsen inflammatory bowel disease, negatively modify intestinal flora, and impair immunity. In addition, the intake of larger quantities of beverages containing BVO is known to cause headaches, irritation of the mucous membranes and skin, fatigue, and memory loss. In July 2024, the FDA withdrew its approval for using bromine (and BVO) as a food additive [61]. Despite evidence and pressure from the interest groups, the FDA is reluctant to remove many of the mentioned additives from food. To avoid this, a new FNA must have clearly outlined policies that are publicized.

### 2.3. Minimizing Harmful Effects from Smoked and Preserved Meat

Ham, bacon, and smoked meats are consumed widely in the American diet, yet they contain under-appreciated harmful additives that pose significant health risks. One of the primary concerns is sodium nitrite, a preservative used to maintain the color and extend the shelf life of processed meats [62]. When heated, nitrites in ham and bacon can react with amines in the meat to form nitrosamines [62,63], which have been identified as potent carcinogens linked to colorectal and gastric cancers [64,65].

Similarly, smoked meats contain carbonyl compounds, such as formaldehyde and acrolein [66], which are formed during the smoking process [67]. They are known to cause significant oxidative stress, inflammation, and DNA damage, contributing to carcinogenesis [68]. Acrolein is a similar major carcinogen present in tobacco [68]. These findings highlight the need for greater awareness of the dietary risks associated with processed and smoked food, namely meats, reinforcing the importance of consuming fresh, unprocessed alternatives to reduce long-term health risks [66], also present in red and processed meat [69].

## 3. Evaluations of Food and Nutraceuticals and Evidence-Based Medicine

Many innovative therapies, as well as repurposing already approved agents, originate from clinical observations and university-led research based on smaller, patient-oriented, prospective, and ecological trials. However, the FDA and the pharmaceutical industry disregard these favoring patented medications [70,71]. This is primarily due to the misconceptions perpetuated by the pharmaceutical industry and conflicted scientists that large randomized controlled trials (RCTs) are propagated as the sole means of establishing a pharmaceutical agent’s efficacy and safety [72]. RCTs were designed initially to obtain drug approvals and have nothing to do with nutrients [16]. It was adapted later by the pharma for nutrients to discredit them and by academics to get funding.

### 3.1. Randomized Clinical Trials Are Not the Right Approach to Evaluate Nutrients

With their substantial financial resources, large pharmaceutical companies can afford extensive RCTs, which are now mandated by the FDA [73,74]. A recent example is vitamin D’s role in various disorders, where almost all recent RCTs suffered from significant study design flaws [71,72,75]. Many of these RCTs are also either directly or indirectly funded by the pharmaceutical companies. Additionally, the industry created and promoted evidence-based medicine (EBM) to reinforce RCT model [76], limiting academic institutions and smaller research entities, such as biopharma, from conducting large-scale RCTs [77,78]. The following section provides details of this.

Ironically, large community-based prospective observational and ecological studies are more appropriate for nutrients than RCTs or Mendelian randomization trials [16,79]. The latter are unsuitable for nutrient association studies, as outcomes are too distant from the datasets; unlike pharmaceuticals, nutrients function as threshold compounds [75]. Researchers have ignored the fundamental differences between synthetic drugs and functional nutrients, leading to costly and poorly designed nutrient RCTs—such as those on vitamin D and omega-3—that failed to meet their primary endpoints—the VITAL study is a classic example. [17,75]. Meta-analyses based on flawed RCTs amplify these errors, with misleading conclusions that the nutrient tested is ineffective [16,17].

Scientists, physicians, and the public should not be swayed by the prevailing emphasis on inappropriate use of RCTs for evaluating nutrients, primarily by industry or the FDA [16,75]. Despite widespread claims, RCTs are unsuitable for evaluating nutrients, and should not be considered the gold standard for assessing food and nutraceuticals [17,74]. This misconception must be corrected [73]. Physicians and healthcare professionals should adopt a more flexible approach in selecting treatments, recommending over-the-counter agents, and incorporating wholesome nutrients based on individual health needs, patient preferences, and robust scientific evidence, particularly from community-based ecological studies [16,75].

### 3.2. Failures of Evidence-Based Medicine

In the early 1990s, Pharma-driven groups created the guidance for EBM concept and related guidances. EBM systems, developed from conclusions drawn primarily from RCTs, Cochrane reviews, systematic reviews, and meta-analyses they all suffer from significant limitations [2]. Consequently, there are major limitations to applying EBM in medical practice. In addition, as with statistics, none of the EMB findings and recommendations apply to individual patients’ circumstances [2]; they were misapplied to clinical practice. Besides, the EBM model depends on the reliability of data from clinical trials, for which RCTs are unsuitable.

In addition to being restrictive in extrapolating to communities, RCTs used for drug approval are exclusively designed and conducted by the pharmaceutical industry [80]. In addition, almost all these industry-conducted RCTs are published in paid journals using the names of academics to attract credibility [76]. Besides these, expanded EBM recommendations by various scientific societies should be considered only as guidance, not mandates, when treating patients. Since they do not apply to individual patients, they should not be used in clinical protocols [77]. In addition, the EBMs are designed to prioritize the use of patented pharmaceuticals. Therefore, it is unsurprising that EMB recommendations increased the cost of care drastically, without improving clinical outcomes. Ignoring these fundamental facts has led to more harm than benefits, which are vivid now in clinical practice worldwide.

Despite the initial hype, EBM has failed over the past few decades due to corporate interests, false propaganda, regulatory failures (i.e., FDA) [35], and the commercialization of academia [81]. The pharmaceutical industry prioritizes shareholder profits over public health [81]. Consequently, underfunded universities and faculty members increasingly rely on pharmaceutical funding, with some faculty members viewing it as essential for sustaining their academic careers [76]. The reliance on pharmaceutical funding by academics continues to increase as the percentage of funding for research from NIH declines.

By accepting pharma grants, endowments, and other corporate contributions, create significant conflicts of interest. As a result, many university researchers compromise their scientific integrity by becoming extensions of the pharmaceutical industry [81]. Even though, EBM may be helpful in population-based strategies like vaccination schedules; but EBM does not provide such. Notably, many innovative therapies originated from clinical observations and university-led fundamental research based on smaller trials, yet the FDA and the pharmaceutical industry often overlook them.

### 3.3. EBMs Add to the Escalating Cost of Healthcare

EBM is designed by groups, exclusively focusing on drugs and devices. This approach has created a vacuum that prevents the application of common-sense decision-making by physicians—prioritizing what is best for an individual patient rather than on a statistical basis for broader population [77]. Strict adherence to rigid EBM protocols forces physicians to follow AI-driven algorithms, neglecting personalized, patient-centered care. These EBM approaches have detrimental effects across most medical fields, contributing to higher emergency room re-admissions, seen across the country.

This rigid setup leaves no room for applying common sense—such as deviating from EBM guidelines to act in the best interest of a patient—due to fear of repercussions. Consequently, holistic and orthomolecular medicine are ignored and nutrition education in medical school curricula is neglected, perpetuating this, imperfect system [77]. Additionally, mandates from hospitals, scientific societies, and regulatory bodies enforcing firm protocol adherence have fostered an over-reliance on EBM-based recommendations, despite their harm. These narrow approaches significantly restrict the physicians’ autonomy in clinical decision-making, limiting their ability to choose the most appropriate investigations and treatments for individual patients [2].

The misconception that RCTs are the sole means of establishing an agent’s or device’s efficacy and safety stems largely from pharmaceutical industry influence under FDA oversight [77]. With vast financial resources, large pharmaceutical companies can afford extensive multi-center RCTs, despite their limitations the FDA now mandates [80]. Additionally, the industry promotes the EBM model to modify clinical recommendations, and, restricts academic institutions and smaller research entities, like biopharma, from conducting large-scale RCTs. Recent examples of vitamin D’s role in various disorders, where nearly all mega RCTs suffered from major design flaws, which should be a concern for the funders and an issue to be critically analyze [71,72,82]. Expanding EBM beyond medications and devices could be beneficial [83]. However, little evidence supports their use in health policies, especially for disease prevention. While EBM may provide average results for heterogeneous patient groups, they are more useful for broad community or national policies than making clinical protocols [2].

### 3.4. RCTs and EBMs Are Not Desinged to Evaluate Micro-Nutrients

Fundamental biological differences between pharmaceutical agents and micro-nutrients render RCTs unsuitable for evaluating their efficacy [84,85,86]. While pharmaceuticals generally produce linear, dose-dependent responses—which fit well for RCT design—micro-nutrients like vitamin D exhibit curvilinear, threshold-based effects [75,78]; significantly different from synthetic drugs. Once the physiological sufficiency is achieved, further increases confer little or no added benefit, another dynamic that RCTs fail to capture [75].

As Heaney and others have emphasized [84], this threshold behavior is combined with biological variability and other inherent factors. These make both RCTs and Mendelian randomization improper (or inferior) tools for assessing micro-nutrient efficacy [84]. Unlike drug trials, constructing a true placebo group in nutrient studies is nearly impossible due to uncontrollable variables such as sun exposure (UVB), dietary intake, and self-supplementation. These factors introduce significant confounders, undermining the internal validity and statistical power of such trials.

### 3.5. Large Vitamin D RCTs and EBM Frameworks Amplify Misleading Conclusions

Recent larger vitamin D RCTs, including VITAL and D-Health, etc., have suffered an array of methodological flaws: enrolling already vitamin D–replete subjects, neglecting to measure baseline or achieved 25(OH)D levels, using subtherapeutic doses, allowing protocol violations, and failing to control for off-trial supplementation [75]. These design errors compromise outcome reliability and diminish translational value [87,88].

Pharmaceutical companies originally developed RCTs to evaluate the efficacy of their synthetic drugs. Commercial priorities shaped their design, making them poorly suited for micro-nutrient research. The rise of EBM further entrenched this bias, promoting patented drugs over affordable, widely available generics and repurposed agents [89,90]. Notably, it has been reported that industry-sponsored evidence is incomplete and biased, while most intervention studies that include data presented to the regulators are industry-sponsored [91]. Consequently, the overall evidence about most interventions is incomplete and biased, and thus untrustworthy (e.g., COVID-19 vaccines and antiviral agents). As a result, patients may be given less effective, harmful, or more expensive treatments with less efficacy [89,90,91].

The reliance on RCTs and EBM in nutrient science without adhering to fundamentals—and even in pharmaceuticals—misapplies methodology and is geared to conform to funding agency mandates—mainly from institutions like the NIH and NSC. This flawed approach, often shaped by commercial interests, distorts scientific inquiry and policy. As a result, the healthcare system prioritizes and encourages the use of expensive treatments over cost-effective generics and repurposed drugs and preventive strategies. This undermines public health and inflates national healthcare expenditures. This practice is also encouraged by scientific societies, the WHO, and the CDC. State Medical Boards add fuel to this by preventing physicians from using generics and repurposed agents (i.e., off-label prescribing of already approved medications) over patented medications, as witnessed during the COVID-19 pandemic [92] and globally [71,72].

In contrast, researchers who apply robust prospective observational studies, ecological analyses, and high-quality meta-analyses (that exclude poorly designed RCTs) consistently uncover clear and more practical evidence about the efficacy, safety, and cost benefits of micro-nutrient interventions [75,93]. These methods better reflect the real-world complexity of nutrient physiology and align more closely with the needs of public health and clinical nutrition.

## 4. Key Reasons for the Escalation of Chronic Diseases

The escalation of chronic diseases can be attributed to several key factors, including poor dietary habits, sedentary lifestyles, and inadequate public health policies. Diets high in processed foods, sugars, unhealthy fats, and additives contribute significantly to conditions like obesity, diabetes, and cardiovascular diseases. Sedentary behaviors, such as prolonged desk and screen time and a lack of physical activity, enhance these risks. Moreover, the lack of emphasis on disease prevention in the healthcare system and the over-reliance on reactive treatments further perpetuate this crisis. Socioeconomic factors, including limited access to healthy food and healthcare, also play a crucial role in the rising prevalence of chronic diseases. Together, these interconnected factors have created a perfect storm for the growing burden of chronic health conditions worldwide.

### 4.1. Compartmentalized Medicine and the Cost of Excluding Complementary Care

Chronic diseases like heart disease, cancer, and type 2 diabetes have surged in recent years, largely because the current healthcare system is designed to treat symptoms instead of identifying and eliminating the root causes. This symptom-focused, specialist-driven, compartmentalized approach often leads patients through multiple specialists—cardiologists, endocrinologists, oncologists, etc., like on a conveyor belt, each charging patients, which bloats healthcare costs. Aside from generating additional revenue, multiple specialist consultations provide little added value to patients.

One study in Singapore found that per capita healthcare spending rises sharply in a stepwise manner with each additional chronic condition. Individuals with five or more chronic conditions incur USD 14,768 annually, versus USD 1081 for those with none—a 92% increase in the cost of care [94]. This fragmented model not only raises overall costs but also fails to provide holistic, long-term solutions that are not helpful to patients. In general, younger individuals (ages 15 to 35) tend to have lower-quality diets compared to older adults (ages 50 to 70) [95].

Poor diets and sedentary lifestyles serve as primary contributors to the rise of most chronic diseases [96,97]. Nevertheless, they remain underemphasized and unattended in clinical practice and insufficiently addressed in public health policy [41]. Observational data indicate that adherence to diverse, whole-food-based dietary patterns—such as the Mediterranean or prudent diet alone—can reduce the risk of cardiovascular disease, neurodegeneration, and cancer by 6–13% [94,98].

### 4.2. Root Causes vs. Symptoms: Systemic Failures Fueling Chronic Disease and Costs

Traditional healthy diets share common features that holistically help human beings and their health. Although average Western diets and fast food are unhealthy, alternatives are available through vegetables and fresh fruits, whole grains, legumes, and nuts, in conjunction with less highly processed foods—animal-based foods and processed meats [99]. Epidemiologic studies and clinical trials have provided certain types of diets that reduce risks of non-communicable diseases (NCDs) [100], such as cardiovascular diseases, cancer, and obesity [101], that contribute to 50% of deaths in Western countries [41,102].

When healthy diets are integrated with beneficial lifestyles, the incidence of NCDs is reduce significantly [41]. Sedentary behavior [96] alone is reported to contribute approximately GBP 0.7–0.8 billion annually to NHS costs in the UK [96]. Implementing healthy diets and increasing physical activity on national and global scales will improve the health of millions, significantly reduce the incidence of NCDs and preventable deaths, and lower overall healthcare costs [96,97,102,103].

Rather than relying on pharmaceuticals, the utilization of effective public health strategies, like promoting regular physical activity, improving dietary quality, eliminating harmful foods, and supporting mental health, financial, and regulatory frameworks enable them to significantly reduce the impact of NCDs—the incidence of chronic diseases and healthcare expenditure [102]. In addition, a Lancet review concluded that population-level interventions like taxation of unhealthy foods, subsidizing fruits, vegetables, and nuts [99], and enhancing nutrition labeling could not only improve health outcomes but also be cost-saving by offsetting the future costs of chronic care, as well as opportunity costs [100,104]. Additionally, a systematic review suggests that physically active individuals enjoy better life, significantly lower healthcare costs due to reduced incidence of chronic conditions—though an aging population may offset some of these reductions [100,103,104].

Chronic diseases—such as type 2 diabetes, obesity, cardiovascular and pulmonary conditions, arthritis, and osteoporosis—are on the rise due in large part to poor diet, unhealthy lifestyle choices, and over-reliance on medications and compartmentalized medical care. Suboptimal nutrition alone drives approximately USD 50 billion annually in U.S. cardiometabolic-related healthcare expenditure and contributes to 20% excess deaths from heart disease, stroke, and diabetes [105]. When combined with physical inactivity and elevated body mass index, these conditions multiply healthcare costs. For example, obese individuals incur 36–44% higher medical expenses, while physically inactive adults face 24% higher treatment costs [106].

### 4.3. Over-Medicalization Escalates Ill Health and Healthcare Costs

The deterioration in public health initiates a cycle of medicalization [106]—frequent consultations, prescription medications and associated adverse reactions, excess diagnostic tests, hospitalizations, and therapy sessions—all of which escalate healthcare expenditure. In 2010, approximately 4% of U.S. healthcare expenditure (about USD 77 billion) was due to medicalized conditions (e.g., obesity, sleep disorders, etc.), exclusive of the broader chronic disease burden [101]. In addition, there is considerable evidence that those with chronic diseases, despite multiple medications, have poor adherence to therapy, making the vicious cycle worse [105]. Evidence shows that individuals with chronic ailments spend, on average, more than USD 2200 extra annually in total healthcare costs and an additional USD 1000 on hospital stays, highlighting the economic burden of symptom-based care rather than root-cause treatment, as illustrated earlier [106].

To reduce both the human and financial toll, healthcare systems must pivot from over-medicalization [106] toward holistic prevention, leveraging nutrition, physical activity, and public health initiatives. Since the author converted his academic medicine/clinical practice thirty years ago to encompass holistic and orthomolecular medical practices, for each prescription that the author wrote, he managed to take off eight prescriptions. The discontinuation of prescription medications alone made his patients feel and become better physically, needing fewer clinic visits. This transition from treating symptoms with medications to a root-cause-driven medical approach could alleviate the burden on clinics and reduce unnecessary medical expenditure while improving immediate and long-term patient outcomes and overall societal well-being.

### 4.4. Early Correction of Micro-Nutrient Deficiencies Reduces the Incidence of Chronic Diseases

Early and targeted micro-nutrient supplementation can significantly alter the course of disease progression when administered at the onset or during the subclinical phase. However, healthcare professionals often overlook this fundamental principle. For instance, an RCT combining vitamin D_3_ (2000 IU/day) with vitamin K_2_ (240 µg/day) over 24 weeks showed a 7.1% reduction in post-COVID syndrome in adults compared to the control group. It also reduced inflammatory markers such as sTNF-R1 and oxidized LDL, highlighting the rapid anti-inflammatory and antioxidant benefits of vitamin D (albeit the vitamin D dose used is less than a third of what is needed) [107]. Similarly, a systematic review of micro-nutrient supplementation—including vitamins C and D and zinc—demonstrated reduced incidence and severity of acute respiratory infections, supported by strong immunomodulatory effects during early supplementation that costs minimal [108].

These findings suggest that early micro-nutrient intervention mitigates acute inflammatory insults—as in post-viral syndromes, plaque destabilization, and atherosclerosis—interrupt the progression of NCDs. Many chronic conditions begin in adolescence and progress silently into adulthood. Initiating supplementation during these critical periods can offer lasting benefits and significantly reduce long-term healthcare burdens and costs.

### 4.5. Lack of Actions to Control Chronic Diseases

Chronic diseases, including cardiovascular conditions, cancer, diabetes, and chronic respiratory diseases, account for over 60% of global deaths and constitute the majority of healthcare expenditures [109]. Without decisive intervention, the proportion of deaths attributable to chronic diseases is projected to exceed 75% by 2030 [110,111], resulting in an estimated additional $5 trillion in healthcare costs. It is time to act to prevent such an escalation of costs. The primary contributors to these conditions include unhealthy lifestyle choices, such as poor diets [3,5], physical inactivity, and behavioral patterns exacerbated by the systemic neglect of preventive measures [4,28]. A comprehensive structural reform in healthcare is imperative to mitigate this crisis and ensure national well-being—inaction is not an option.

By contrast, the proper policies and action implementations can reduce maternal and infant mortality and all-cause mortality [25], increase longevity with health span [9]. However, governments continue allocating most healthcare funding to acute care and symptomatic treatment, ignoring the preventive strategies’ transformative and regenerative potential [28]. By redirecting resources toward prevention, governments could implement cost-effective solutions that improve public health outcomes, significantly reducing healthcare costs, and building healthier populations [27].

### 4.6. Social and Societal Factors Are Not the Main Cause of Rising Healthcare Costs

The current healthcare model disproportionately prioritizes acute care, influenced by lobbying from insurance companies, healthcare corporations, hospital systems, politicians, and the FDA. The global economic burden of chronic diseases is expected to reach approximately $47 trillion by 2023 [5], with chronic conditions accounting for 75% of worldwide deaths by 2030 [109,111]. There is an alarming trend in the exacerbation of social determinants of health, including economic disparities, limited access to nutritious foods, unaffordability, and inadequate health education [6].

The neglect of social determinants of health, nutrition, and effective public health measures continues to fuel this crisis [31,110]. Despite claims, this is not a primary reason for the escalating healthcare costs. Despite massive healthcare expenditures in the United States, which surpass those of all other nations, there has been no commensurate improvement in the quality of care [7,30,112,113]. The U.S. healthcare system has deep-rooted problems. As a result, merely tweaking the ACA or adjusting rules (as in the FDA) will not significantly reduce costs unless the underlying causes are directly addressed.

The consequences of this neglect are severe—rising hospitalizations, increased maternal and childhood deaths, more premature deaths, escalating healthcare costs, and surging insurance premiums [6,104]. Additionally, unethical incentives to hospitals and physicians and overprescription practices contribute to widespread polypharmacy. This leads to adverse drug reactions, harmful interactions, increased hospitalizations, preventable deaths, and poor clinical outcomes [7]. These outcomes emphasize the urgent need for systemic transformation in the healthcare system to prioritize prevention and address the root causes of chronic diseases [12]. Similarly, for several reasons—chief among them, overly broad mandates—the FDA has also failed to regulate and oversee food and drug safety effectively.

### 4.7. Declining Scientific Output at the NIH and NSF-Funded Research

**Figure 1:**
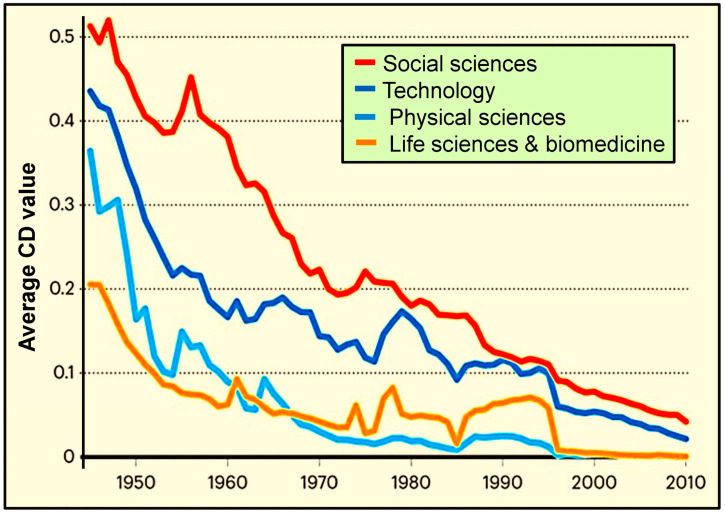
Disruptive Science Index—Illustrates marked reductions in the effectiveness as indicated by the lowering of CD (*y*-axis) of NIH research grants over the past 40 years. Higher CD values indicate more innovative (disruptive) outcomes beneficial to the nation [117] (modified from Kozlov, M, 2023; Nature: DOI: 10.1038/d41586-022-04577-5 (https://barsoom.substack.com/p/dieing-academic-research-budgets, accessed on 23 February 2025) [118].

The National Institutes of Health (NIH) was established in 1948 [114], and the National Science Foundation (NSF), established in 1950 [115], has long funded biomedical research. However, their effectiveness in supporting groundbreaking science that benefits the public has declined due to shifting internal policies and priorities. Research impact is typically measured by the CD (convergent disruption) index [116], where higher values (approaching 1.0) indicate more innovative, disruptive outcomes beneficial to the nation [117]. Alarmingly, between 1950 and 2010, the CD index for NIH-funded research plummeted by 88% in social sciences, 42% in technology, 82% in physical sciences, and 93% in life sciences—yet funding priorities remain unchanged [116]. Figure 1 displays the average CD (disruptive) index of NIH-funded research papers, five years post-publication, ranked on the vertical axis from most beneficial (1) to the least (0).

### 4.8. Examples of Ineffective Mega Grants Awarded to Leading Universities

Over the past two decades, the NIH has allocated substantial research funding to multi-year, high-cost program grants that, in many cases, have not demonstrated cost-effectiveness. Additionally, large single-center grants—some exceeding $40 million [75,78], such as those awarded for the VITAL study [119,120,121], appear to have been influenced not solely by scientific merit but also by institutional prominence and existing funding capacity. This approach has inadvertently disadvantaged smaller institutions. Moreover, recent evaluations have raised concerns about the suboptimal quality of research outputs associated with these multi-million-dollar grants, particularly concerning study design, resulting in the inefficient use of significant taxpayer resources [116,122,123].

Negative findings from several NIH-funded studies have raised concerns, particularly because a substantial portion of the funding was allocated to researcher salaries, leaving limited resources for conducting essential research. Additionally, many large single-center nutrient RCTs—such as the WHI and VITAL studies—were affected by avoidable yet significant design flaws. These included enrolling participants who were already sufficient in vitamin D, failing to confirm baseline vitamin D deficiency [25(OH)D] levels, inherent design biases, inadequate dosing, short study durations, allowing participants to take OTC nutrient supplements, and infrequent vitamin D administration, often at intervals exceeding one month. Appropriate oversight from the NIH, data safety monitoring boards, and the application of stricter scientific rigor could have prevented these methodological shortcomings and yielded more meaningful and reliable results [124,125].

Instead of prioritizing funding for innovative, risky, and scientific breakthrough grants, the NIH and NSF have increasingly diverted funding to mundane, low-impact studies and ideologically driven projects [126], especially those aligned with diversity, equity, and inclusion (DEI) initiatives. Each institute allocates around two billion dollars annually to these secondary efforts [116]. These wasteful trends have diminished research impact, quality, investment value, and societal benefit [127]. Rather than funding based on scientific merit, technological potential, and community relevance, the system favors the status quo and Ivy League institutions [127]. In addition, the NIH has developed a habit of funding projects with completed data at submission [116]. Private-sector investment is gradually filling the gap left by reduced NIH and NSF funding [117], resulting in steady progress but often producing compromised research and falling short of delivering transformative breakthroughs such as the Internet, AI, or machine learning.

### 4.9. Misaligned Priorities and Inefficiencies in Federal Research Funding

Most scientific breakthroughs stem from creative, high-risk proposals exploring novel, cross-disciplinary insights [116]. In contrast, NIH funding often supports multi-million-dollar, renewable grants awarded to established institutions for routine, redundant, or previously conducted research that reinforces the status quo [127]. The inefficiency and inferior quality of work are evident, as only 55% of NIH-funded studies are replicable, especially from large universities where publication volume has risen without corresponding quality gains [127]. Additionally, excessive institutional overheads—ranging from 45% to 78% on top of grant funds that go to administrations—divert resources from the core research of other entities. Capping overheads uniformly at 15% would free up funds substantially to support genuine, competitive R01 grants and other scientific projects at smaller universities and colleges.

Additionally, most NIH and NSF grant budgets are deliberately inflated by investigators in anticipation of funding cuts, resulting in significant inefficiencies and waste. [127]. Reviewers must scrutinize grant proposals more rigorously, and decision-makers must adjust budgets according to actual needs. Institutional directors and staff are responsible for approving funding and must manage and allocate research funds transparently and be held accountable for their decisions [126]. Accountability and transparency are crucial to ensure efficiency and alignment with the core mission of advancing science and technology for national benefit [128]. Figure 1 highlights the sharp decline in practical value (as reflected in the CD index) and impact of NIH-funded research output over the past four decades.

### 4.10. The Affordable Care Act—Pros and Cons

**Figure 2:**
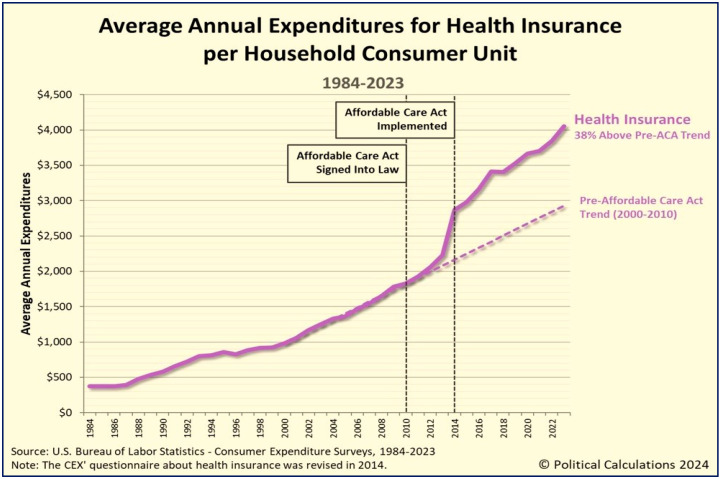
Escalated healthcare costs since the implementation of the ACA (from Political Calculations, 2024; Source: US Bureau of Labor Statistics-Consumer Expenditure Surveys, 1984–2023). The deflection point is after the ACA [128,134].

The Affordable Care Act (ACA), enacted in 2010, was intended to expand health insurance coverage, including for individuals with pre-existing conditions, reduce healthcare costs, and improve quality of care [129]. However, the ACA fundamentally failed to streamline care delivery, improve clinical outcomes, or reduce long-term healthcare costs [130,131]. Over the past decade, the policy trajectory has increasingly been driven by commercial interests with minimal regard for patient well-being, resulting in continuously rising insurance premiums and out-of-pocket expenses for individuals and families [132,133]. Since the implementation of the ACA in 2014, healthcare costs have continually surged [134] (Figure 2), while the quality of care and patient outcomes have declined [113].

As illustrated in Figure 2, although insurance coverage has expanded, it has come at the cost of soaring premiums (subsidized by private insurers), restrictive provider networks, and unaffordable deductibles—issues that particularly affect the middle class [131]. The ACA’s structure disproportionately benefits large insurance corporations and hospital conglomerates, accelerating healthcare system consolidation and sidelining primary care providers [129,132]. Additionally, its administrative complexity has increased overhead costs while yielding limited improvements in overall health outcomes [132].

### 4.11. The Affordable Care Act—Why Should It Be Replaced

Rather than improving access and care quality, the ACA entrenched a commercially driven healthcare model emphasizing revenue generation over patient treatment [131]. For example, hospital systems adopted electronic platforms such as EPIC [132], which are optimized for billing and coding rather than enhancing clinical decision-making or continuity of care [130]. The ACA’s emphasis on standardized cost-containment protocols led to substituting experienced physicians with mid-level providers, thereby increasing fragmentation, inefficiencies [129], and hospital readmissions—particularly through the most expensive emergency departments [133].

Moreover, the hospital software associated with ACA implementation—such as EPIC—prioritizes billing and point-of-service logistics but not on actual patient management and care [130]. While urgent care facilities have flourished under this system, they often operate with fragmented, uncoordinated records that hinder timely and effective treatment with abundant duplication [132]. Meanwhile, the ACA-directed changes have gradually replaced physicians and nurses with “providers” tasked more with robotic triage than treatment, contributing to persistently high emergency room readmission rates [133]. Though profitable for hospitals, this inefficiency burden patients, the federal government, and taxpayers.

In practice, the ACA has favored hospital conglomerates by creating administrative job programs staffed with less-trained personnel while marginalizing primary care physicians—professionals essential to delivering comprehensive, patient-centered care and keeping the cost down. These systemic deficiencies underscore the need to replace the ACA or undergo comprehensive reform to serve society’s needs truly—superficial adjustments will not suffice. ACA replacement must prioritize prevention, coordination, transparency, new technology including AI, ML, and cybernetics, and patient-centered outcomes while reducing waste, inefficiency, and healthcare costs.

### 4.12. Sustainable Modification of Lifestyles

Negative social determinants of health are derived primarily from unhealthy lifestyles and socio-economic disparities [3,5]. However, the current disproportionate funding favors hospital medicine over chronic disease prevention, the main driver for hospitalizations and escalating healthcare costs [6]. Influential lobbying exacerbates this imbalance, leaving chronic diseases, malnutrition [135], and other key social determinants largely unaddressed [31,110]. Despite the high per capita healthcare spending in the USA care quality and clinical outcomes remain stagnant [30,112].

This neglect enhances ill health and increases hospitalizations, premature deaths, and soaring insurance premiums. These indirectly benefit healthcare entities like specialty pharma [136] and pharmaceutical and insurance companies, but at the expense of the public [6]. While the drug costs add a bit, hospitalizations, physicians’ charges, and Medicaid and Medicare costs are towering for the same reasons [6]. Additionally, unethical incentives and overprescribing—especially of patented drugs—contribute to polypharmacy, resulting in adverse effects, preventable deaths, and poor clinical outcomes. Transforming the healthcare system is essential to addressing these challenges at their root.

While the imbalance of high-cost and less-than-optimal services to people is increasing [6], influential healthcare- and insurance-related lobbying aids the neglect of treatable and preventable chronic diseases [137], which exacerbate chronic diseases, malnutrition, ill health, and other social determinants of health [31,110]. Despite the very high per capita healthcare spending in the USA, care quality remains stagnant, and clinical outcomes stay poor [30,112,138]. Fundamental changes in the case system are warranted to reverse this. This neglect fuels increased hospitalizations, fatalities, and soaring insurance premiums. Meanwhile, alongside the devastating opioid and fentanyl crisis, overprescribing and polypharmacy contribute to debilitating adverse effects, a sharp rise in preventable deaths (the highest number of preventable deaths), and poor clinical outcomes [139]. Transforming the healthcare system is essential to resolving these challenges.

## 5. Wastage, Abuses, and Fraud in the Healthcare System

Wastage, abuse, and fraud in the healthcare system are significant issues that drain resources, compromise patient care, and inflate healthcare costs. Wastage occurs when medical supplies, medications, and equipment are used inefficiently or discarded prematurely. Abuse involves misusing healthcare services, such as overprescribing medications or unnecessary medical procedures. Fraud includes intentional acts, like billing services not rendered, falsifying patient records, or submitting false insurance claims. These practices undermine the integrity of the healthcare system, divert funds from essential care, and contribute to the rising financial burden on both the public and private healthcare sectors. Addressing these issues requires stronger oversight, improved accountability, and a culture of transparency.

### 5.1. The Importance of Early Identifying Major Frauds and Abuses in Healthcare

Medicare and Medicaid are among the largest entitlements in the USA; however, funding for Medicare is predicted to be exhausted by 2036 [140]. Fraud and abuses in this healthcare system are documented; studies over the past decades have confirmed over $100 billion in fraud annually [141]. As part of reorganizing the US healthcare system, it is crucial to scrutinize the eligibility criteria and revamp the system; this would include the food stamp program. DOGE and AI systems [38] can greatly aid in eliminating fraud within Medicare and Medicaid programs by identifying and removing fraudulent beneficiaries and enabling legal action against those illegally receiving federal funds. Such a waste of taxpayer money is estimated to exceed $200 billion annually. The government must reinvest these savings to preserve the long-term sustainability and solvency of essential programs for seniors and low-income individuals [142]

Long-term care and disability programs, including Social Security Disability Insurance (SSDI) and Veterans Affairs (VA) healthcare services, are inefficient and contribute to excessive healthcare spending [143]. They are also known to have significant waste, abuses, and fraud [144]. These programs are known to have embedded improper enrollment of beneficiaries, leading to misallocation of resources and unnecessary expenditures. The VA system, for example, is top-heavy and has been criticized for high administrative costs, long wait times, lack of reasonable access, and mismanagement of resources, leading to delays in patient care and poor clinical outcomes [145,146]—another area that must be fixed.

Additionally, SSDI is highly vulnerable to fraud, with some beneficiaries remaining on disability for years—even lifelong—despite being medically fit to return to work. For example, a RAND Corporation report found that 10–20% of SSDI recipients are capable of working and could be reintegrated into the workforce with appropriate vocational support [147,148]. Improving accessibility, reforming eligibility criteria, implementing stricter audits, and investing in preventive care could significantly reduce waste in these programs while improving patient outcomes [145,146].

### 5.2. Key Programs with the Highest Wastage of Healthcare Funds in the USA

Substantial inefficiencies burden the U.S. healthcare system, with major entitlement programs contributing heavily to financial waste and abuse [149,150]. Medicare and Medicaid—America’s most extensive publicly funded healthcare programs—are key sources of this inefficiency, driven by fraud (claims from non-existent recipients and providers), administrative overhead, unnecessary procedures, and improper provider payments [141]. In 2023 alone, Medicare spent an estimated USD 1.0 trillion to serve around 66 million elderly and disabled individuals, processing over a billion healthcare transactions [151].

The lack of integrity in U.S. government-controlled healthcare agencies poses a serious concern. Fraudulent Medicare claims and inappropriate Medicaid program enrollments are estimated to exceed USD 250 billion annually [152]. Whereas the federal agency, the Centers for Medicare & Medicaid Services (CMS) in the U.S. Department of Health and Human Services (HHS), CMS-Center for Program Integrity (CPI), responsible for combating fraud, waste, and abuse in Medicare, only reported USD 14.5 billion in losses due to fraud [151]. Meanwhile, the Department of Government Efficiency (DOGE) identified over USD 140 billion in improper payments by CMS in 2024 [153]. These figures highlight the urgent need to eliminate fraud and abuse of healthcare funds within government agencies.

Medicaid is a joint federal-state program that finances health care for low-income and medically needy individuals. It is the second most costly healthcare entitlement program. In 2023, it incurred an estimated $849 billion in combined federal and state spending to provide services to approximately 90 million individuals [141], including undocumented immigrants. A Government Accountability Office (GAO) report estimated that improper payments within Medicare and Medicaid alone exceeded $100 billion annually [141].

Nevertheless, neither the federal nor the state governments have made serious attempts to stop these irregularities. Although there were some uncoordinated efforts in the past by the Centers for Medicare & Medicaid Services (CMS) and the Department of Health and Human Services (HHS) to address inefficiencies, these measures had minimal impact [141]. Without relying on temporary fixes, a comprehensive restructuring of this entitlement program and the ACA—is warranted to enhance efficiency, eliminate waste, reduce fraud, and ensure long-term sustainability.

### 5.3. Federal Healthcare Losses Due to Fraud and Waste

A major contributor to this waste is fraud, including fraudulent billing, upcoding, and phantom claims, all of which significantly drive-up costs [154]. Additionally, administrative complexity—stemming from fragmented systems, overregulation, unnecessary documentation (i.e., excessive paperwork), and duplicative services—wastes physicians’ time and inflates expenditures without providing any added benefit. Administrative costs in U.S. healthcare account for approximately 25% of total spending, compared to just 10% in other high-income countries [149,150].

The largest component of healthcare “administration” does not involve essential tasks such as scheduling or supply management, but rather activities centered on securing reimbursement from third-party payers, including government programs and private insurers. A substantial portion of physicians’ (PCPs) time is consumed by compliance, securing insurance approvals, and documentation requirements, often equaling the time spent on direct patient care. Despite this, such burdens are not billable or typically not categorized as administrative overhead. This excessive non-clinical workload is a major contributor to physician burnout. Addressing these inefficiencies requires policy reforms to streamline billing processes (and using AI), reduce top-heavy administrative staffing, enhance early fraud detection (cybernetics), and eliminate unnecessary bureaucratic burdens [154].

When hospitals and healthcare providers/doctors are paid by insurance companies or the government, there are a few checks. These entities dramatically inflated billings, escalating healthcare costs (a self-sustaining Medical Industrial Complex, MCI). In addition, intermediaries and suppliers also unscrupulously inflate the costs (discussed below). These costs are passed on to the consumers as insurance premiums, copays, etc. Therefore, unsurprisingly, healthcare costs in the USA are the highest in the world, but not the quality.

Another major area of healthcare fund wastage stems from overutilizing unnecessary medical tests and treatments, often driven by defensive medicine and financial incentives within the fee-for-service model [150]. Studies indicate that up to 30% of US healthcare spending is wasteful, with excessive diagnostic imaging, redundant lab tests, and unnecessary hospitalizations contributing significantly [150,155]. Defensive medicine (and the lack of familiarity and not using common sense), where physicians order extra tests to avoid potential malpractice lawsuits, further inflates costs without improving patient outcomes [155].

Furthermore, pharmaceutical expenditures are disproportionately high due to inflated drug prices and the overprescription of brand-name medications when cost-effective generic brands are available. A study has reported that excessive drug spending costs the US healthcare system approximately $200 billion annually [156]. Implementing value-based care models, incentivizing the use of generic brands, orthomolecular and holistic medicine, and promoting common-sense clinical guidelines could help reduce such inefficiencies.

### 5.4. Taking Decisive Steps to Reverse the Current Negative Trend

The FDA’s mission and responsibility is to protect the public by ensuring the safety, efficacy, and security of human and veterinary drugs, biological products (including vaccines), and medical devices, and by ensuring the safety of the nation’s food supply, cosmetics, and products that emit radiation [157]. The FDA is also responsible for advancing public health by enabling speed innovations that make medical products more effective, safer, and affordable. The public also relies on the FDA to obtain accurate, science-based reports on medical products and foods to maintain and improve the population’s health [157]. While innovative healthcare is important, safety is the most important responsibility of the FDA. However, when a significant proportion of the drugs approved by the FDA had major adverse effects, it led to a loss of trust in the FDA [158].

Urgent reforms and measures to restructure the FDA are required to reverse the current negative trend, to improve health outcomes, and to improve the nation’s well-being. In the USA, the current profitable US sick-care model prioritizes treatment over prevention, which allows for worsening chronic disease burden and costs, thereby creating a perfect storm, and underscoring the need for immediate change [159,160]. The FDA’s inefficient oversight of food and over-the-counter products is not helping the nation’s health or progress in public health [161].

### 5.5. Pharmacy Benefit Manager System—The Need for Abolishing It

Prescription drug costs continue to rise for multiple reasons, including the exploitation of the “safe harbor” provision in the Medicare Anti-Kickback Statute by Group Purchasing Organizations (GPOs) and Pharmacy Benefit Managers (PBMs) [134]. PBMs are large conglomerates that manage prescription drug benefits for health insurance plans [162,163]. As intermediaries between insurers, pharmaceutical manufacturers, and pharmacies, PBMs wield considerable influence over drug pricing and access [134,164]. The PBM industry is dominated by three major players—CVS Caremark Rx, LLC (a division of CVS; Woonsocket, Rhode Island), Express Scripts Inc. (a division of Cigna: St. Louis, Missouri), and OptumRx Inc. (a unit of UnitedHealth Group; den Prairie, Minnesota) [165]—these are operating with limited transparency [162,164].

These entities impose prior authorization requirements and set reimbursement rates that often favor insurer cost savings over patient care [166]. Using their substantial market influence [165], PBMs inflate drug prices and restrict access to essential medications, driving up overall healthcare costs [164]. The Department of Health and Human Services (HHS) should urge Congress to repeal 42 U.S.C. § 1320a-7b(b)(3)(C) and related provisions such as 42 C.F.R. § 1001.952(j), which currently shield improper kickback arrangements. According to Physicians Against Drug Shortages, PBM-related unethical practices are estimated to cost consumers over USD 100 billion annually [134]. Given their redundancy and harmful effect on affordability, access, and consumer costs, dismantling the PBM system and enforcing robust anti-corruption measures are necessary [128,134]. These reforms would significantly lower healthcare expenses and simplify prescription drug access for both patients and providers [165,166].

### 5.6. Behind the Pharmacy Counter: How PBMs and GPOs Inflate Drug Prices

Because of these two entities, patients now endure higher deductibles and frequent denials of medications and procedures, and authorizations, while physicians face yearly payment reductions, increasing administrative costs, and mounting regulatory burdens. The beneficiaries are large managed-care conglomerates, and the intermediaries that profit at multiple levels. PBMs and Group Purchasing Organizations (GPOs), in particular, control drug formularies, decide which medications are covered, and receive rebates—essentially kickbacks—from manufacturers and insurers.

Due to price manipulation, it is ironic that the self-pay price at retail pharmacies is lower than the insurance copay for certain medications. While the cost of many items is capped through the insurance plans, the same holds for imaging studies like X-rays, which can be more affordable and provide quicker access for cash-paying patients. Other cost-effective options include joining medical cost-sharing organizations, consulting physicians outside government or third-party contracts, and enrolling in Direct Primary Care (DPC) practices. The public should push for regulatory and legislative reforms through Congress and the HHS to broaden alternatives to ACA coverage and expand the use of medical savings accounts.

## 6. Strategic Solutions to Curb Escalating Healthcare Costs

Hospitalization, especially the utilization of Emergency Rooms (in most countries, this is called accident and emergency services), is the most expensive healthcare system [3,20], costing approximately 80% of the cost of care [21]. Chronic diseases predominantly drive this, resulting in an average adjusted cost of $14,200 per inpatient stay at community hospitals in 2019, which is doubled in larger hospitals [21]. Inpatient costs continue to escalate, as do health insurance premiums, regardless of the Affordable Care Act [19]. Despite the vast per capita and total healthcare expenditures incurred by the US, healthcare delivery and quality are stagnant [9,14].

### 6.1. The Lack of Oversight of Healthcare Regulatory Enforcement

The lack of oversight and regulatory enforcement has enabled the widespread marketing of contaminated and harmful food nationwide. Watchdog organizations have reported that despite being aware, the Center for Food Safety and Applied Nutrition (CFSAN), the food regulatory division of the FDA, has consistently failed to take timely action on critical safety and public health concerns. These include pathogens in irrigation water used for production, heavy metal contamination in baby foods, and other hazardous contaminants [167]. Furthermore, the FDA has largely overlooked numerous chemicals of concern, such as per- and poly-fluoroalkyl substances (PFAS), commonly known as forever chemicals, which are prevalent in the food supply and packaging [168].

While the FDA has spent over a decade working with companies on voluntary sodium reduction, many other nations have successfully implemented stringent regulations to address this issue years ago [168]. Despite multiple updates to food safety regulations, the CDC estimates that over 125,000 people are hospitalized, and about 3000 die annually due to foodborne illnesses [167]. The recent outbreak linked to contaminated infant formula is a stark reminder of the devastating consequences of failing the FDA-managed food safety system [167]. Without immediate and comprehensive reforms, public health will continue to suffer from preventable foodborne illnesses and toxic exposures.

### 6.2. What Needs to Be Achieved

Immediate measures should prioritize healthier food choices, stricter safety standards, reduced food processing, and the promotion of better personal health habits. Restructuring the FDA and reforming or replacing the Affordable Care Act (ACA) are crucial. These changes should shift the focus toward a prevention-based healthcare model, which is essential for building a sustainable and effective system [27]. This is critical for building a sustainable and effective system. Simultaneously, as mentioned, Congress must investigate and initiate FDA restructuring to enhance efficiency and public health outcomes.

Separating the regulation of drugs and nutraceuticals is not new; it is practiced successfully in Serbia and represents a logical and efficient model. The Medicines and Medical Devices Agency regulates medicines and medical devices in Serbia, while two ministries manage food and dietary products. Similar frameworks exist in Costa Rica and other nations, albeit with variations. Proposing a split of the FDA’s broad but often inefficient authority over both sectors is unique to the U.S. However, it could be a model for enhancing efficiency, accountability, and public safety worldwide.

Addressing these challenges requires coordinated actions from the government, the healthcare system, and stakeholders. Policy reforms and enacting new laws are crucial for a shift to a prevention-focused healthcare system [157]. Prioritizing health literacy and education at all levels to empower individuals (the public) with knowledge about proper nutrition, including functional nutrients and healthy lifestyle choices, and ensuring access to affordable [4] nutritious food is essential in reducing health disparities and enabling people to maintain good health.

As discussed, chronic diseases are responsible for most hospital admissions and healthcare costs. These cannot be managed effectively through symptomatic treatment alone or by tinkering with current policies without addressing the root causes. Addressing their root cause as a cluster, rather than targeting each chronic disease, is vital for achieving meaningful, long-term health improvements at a lower cost [12].

Overburdening healthcare workers with excessive regulations, paperwork, and intermediary exploitation undermines morale, stifles innovation, restricts prescribing independence, and hinders patient management. These barriers limit access to preventive care and drive-up healthcare costs. Reforming policies to prioritize science-based, patient-centered approaches that restore physician autonomy is crucial for enhancing outcomes and reducing expenditure.

### 6.3. The Ongoing Conflicts of Interest in Healthcare Agencies

Financial ties between FDA officials and the pharmaceutical industry have undermined public trust [169]. Despite disagreements over fragmented and insufficient evidence, these excessively close associations between FDA and CDC directors and pharmaceutical company sponsors the approval of drugs suggest undue influence in the FDA’s decision process [170,171]. In addition to barring the current too-close associations, it is essential to have stricter policies on post-tenure pharma employment prohibitions, which are necessary for impartiality and to regain the lost public trust [169]. These are essential to restore credibility of the FDA and the trust of the public.

Ensuring transparency in drug approvals remains a critical issue, with concerns about the FDA approving drugs based on borderline results, tolerating protocol violations in clinical trials, and accepting data with inadequate statistical power from pharmaceutical companies [73,158]. Additionally, the denial of drugs that should have been approved due to conflicts of interest within the industry has raised additional ethical concerns [35,172]. Close associations between FDA officials and pharmaceutical sponsors as well as disagreements over insufficient evidence, suggest undue industry influence on the approval process [159,173].

The FDA’s persistent lack of transparency during drug evaluations and refusal to allow independent scientists to review original clinical trial (RCT) data, safety, and efficacy (as with the COVID-19 vaccine) has eroded its credibility and trust [171]. This oversight is crucial for physicians and patients to identify biases and make informed decisions [157]. However, superficial reforms, claims without substantive changes, will not address these issues [174]. The Congressional FDA Reform Act of 2012 failed to eliminate conflicts of interest among FDA employees and government agencies [35,159].

Without strict oversight and accountability, the integrity of the drug and medical device approval process remains compromised, undermining public trust. These external factors, often from drug companies or other stakeholders, exercise inappropriate pressure on the FDA staff to approve a drug or medical device, which could potentially compromise the scientific review process [173], and prioritizes commercial interests over public health concerns [172].

### 6.4. The Failures of the FDA to Evaluate and Approve Generic, Early Therapies

In 1994, in response to public outrage over the FDA’s increasingly restrictive actions against natural medicine, the Dietary Supplement Health and Education Act (DSHEA) was passed, effectively limiting the FDA’s authority to regulate naturally occurring supplements [175]. Despite that, the FDA continues to overreach and suppress natural components and therapies, including peptides, function-based phytonutrients, bioactive or nutraceuticals, stem cell therapy, hyperbaric therapies, raw milk, psychedelics, and cost-effective (off-patent) anti-viral agents such as ivermectin and hydroxychloroquine [176].

Natural and unprocessed foods, vitamins, minerals, and nutraceuticals are essential for optimal human health. As per the regulations, these cannot be patented [71]. Consequently, despite the health or medicinal values of some of these, the FDA disregards these widely available over-the-counter agents [176], favoring expensive patented medications, often in alignment with other agencies, such as the Centers for Disease Control (CDC) and the World Health Organization [70,72,73,172]. Specific examples related to SARS-CoV-2 are addressed in the next section.

### 6.5. Rejecting Generic SARS-CoV-2 Treatments Due to Conflicts of Interest

Addressing this issue requires enacting a new Act to eliminate conflicts of interest across government agencies [173]. A major controversy was the approval of COVID-19 mRNA vaccines and antivirals under Emergency Use Authorization despite the availability of cost-effective generic treatments in 2020 [70,71,82,177,178,179,180]. The FDA’s refusal to approve repurposed, effective generic compounds like vitamin D and ivermectin for SARS-CoV-2 [177] led to overwhelmed hospital staff and resources with patients, and unnecessary loss of lives [70,71,72,82]. The DHHS should ensure that such a harmful scenario will not be repeated. Closing regulatory loopholes, eliminating favoritism toward big pharma, and preventing conflicts of interest in the FDA and CDC are crucial [35,172,173].

Cozy relationships between FDA directors and pharmaceutical sponsors and disagreements over insufficient evidence suggest an unwarranted influence on FDA approvals [170,172]. The public considered the non-approval of widely available generics and early therapies against SARS-CoV-2 to be due to conflicts of interest; it had significant negative consequences [71,72]. Policymakers must close these loopholes and enact stronger laws to prevent undue industry influence across the FDA, CDC, and other government agencies [159]. Approvals despite borderline results, tolerating shortcuts in RCTs, and denying economic agents claimed due to ‘insufficient’ evidence further raise concerns of serious conflicts and affect the credibility of the FDA [172]. A new Congressional Act is essential to eliminate conflicts of interest within government agencies [35,170].

## 7. Restructuring of the FDA

The FDA has adopted a structure that is inundated by inefficiencies, conflicts of interest, and regulatory shortcomings that compromise public health. Its dual mandate prioritizes profitable pharmaceuticals over food safety and nutrition policies and neglected preventive health measures, contributing to the higher prevalence of chronic diseases. The DFA’s weak enforcement of dietary standards further exacerbates this issue. Additionally, the revolving door of FDA directors moving into pharmaceutical executive roles raises another major concern lawmakers must address, where financial incentives compromise the FDA’s duty to safeguard public health.

### 7.1. Specific FDA Reforms Are Needed

Internal restructuring of the FDA will not resolve its fundamental issues or major deficiencies. It requires transformative, innovative changes. Key concerns include delays in reviewing drug and medical device applications, reliance on industry funding that creates conflicts of interest, and policies enabling undue pharmaceutical influence over FDA officials must be addressed. In addition, reforms should eliminate rule 340B [181], ensure transparency in the drug approval process, and make clinical trial data accessible to independent scientists [157,171].

Federal Rule 340B (1992) allows hospitals to markup drug pricing, a program designed to support tax-exempt hospitals and clinics for survival in competitive markets [181]. However, this has evolved into a major loophole ripe for abuse. In addition, those manufacturers participating in Medicaid programs provide outpatient drugs to the mentioned entities at significantly reduced prices. By contrast, not-for-profit hospitals significantly increase when they bill the government for funding.

Profits from Rule 340B markups that started at USD 0.4 billion annually now account for over USD 65 billion. Some hospitals charge the full price to vulnerable patients, burying them in medical debt [182]. Such practices were evident during the COVID-19 pandemic, leading to soaring profits—more than doubling profits [182]. These entities inflate costs by exploiting loopholes in Rule 340B to maximize profits while burdening patients, taxpayers, and employers through higher medication prices.

### 7.2. Preventing the FDA-Related Wastage of Funds

Another area in the FDA that needs reform is the 510(k) process, a common path to obtain approval for medical devices [183]. However, the FDA is allowed discretionary influences, hence applying less rigorous reviews of scientific evidence to support their approvals [171]—Another area where conflicts of interest negatively affect the safety and efficacy of approved devices [158,183,184]. While the FDA has claimed that it tightened the procedure of device approvals, the 510(k) process is still not specific, and manufacturers use predicate devices with questionable safety records [158,184].

Reforms must prohibit the industry from incentivizing FDA personnel and replace user fees and perks with a transparent system that eliminates financial conflicts with pharmaceutical companies [35]. Transparency was notably absent during the COVID-19 pandemic concerning mRNA vaccines and antiviral agents, raising concerns about their safety [185].

### 7.3. Regaining Public Trust in Healthcare Regulation

Minor reforms will not restore public trust in the FDA. In addition, a new law is necessary to prohibit the revolving door between the FDA, CDC, NIH, and the private sector, which enables government officials to transition directly into high-level positions within the pharmaceutical industry, creating serious conflicts of interest [172]. The public perceives that FDA directors maintain close ties with major pharmaceutical companies, potentially influencing drug and device approvals in ways that compromise quality and safety.

The public has witnessed FDA directors influencing drug approvals in borderline cases, further eroding the FDA’s credibility [171]. Such practices violate regulatory statutes and public duty, driven by the psychology of gift-giving and fiduciary incentives, ultimately undermining regulatory integrity and trust. These conflicts have been evident in questionable drug approvals, such as the controversial authorization of Aduhelm (aducanumab) for Alzheimer’s and Exondys 51 (Eteplirsen) for Duchenne muscular dystrophy, among others [171,172].

The FDA should be restructured for many reasons, including significant conflicts and its struggle (or failure) to fulfill its primary duty—ensuring drug safety—while failures of overseeing foods, nutraceuticals, and cosmetics require distinct regulatory approaches [170,171]. The FDA takes too long to review drug safety while approving ineffective or unsafe medications and devices [158], raising costs for the pharmaceutical industry and, later, patients. Unlike pharmaceuticals, RCTs or similar regulatory pathways are not the best way to evaluate food and cosmetics [157]. Additionally, the FDA rarely assesses food and nutraceuticals, particularly ultra-processed foods with harmful additives, relying on post-market enforcement after harm has occurred [158].

### 7.4. Lack of Oversight of Government Food Assistance Programs Causing Ill-Health

Research shows that recipients of taxpayer-funded government food assistance programs, such as the Supplemental Nutrition Assistance Program (SNAP), often purchase (using pre-paid cards) high quantities of highly processed, low-nutrient foods, including sugary drinks. These poor-quality diets contribute to metabolic disorders (like obesity and diabetes) [101] and other chronic diseases, raising healthcare costs. The financial burden of these health issues ultimately falls on taxpayers through Medicaid, highlighting the need for policy reforms that promote healthier food choices.

While SNAP cards prohibit alcohol and tobacco purchases, similar restrictions should apply to unhealthy foods like sugary drinks to ensure taxpayer-funded assistance promotes nutritious choices. The Department of Agriculture, in collaboration with the DHHS, should implement this policy. Additionally, they should consider integrating nutritional education to help recipients make healthier dietary decisions. These changes would improve public health, reduce healthcare costs, and align the program with long-term well-being. Since unhealthy foods are often cheaper, promoted by the food industry, and dominate the market, stringent premarket reviews are necessary to protect consumers from harmful, over-processed foods and additives.

In addition, the FDA should ban certain practices, such as limiting vaccine use to the vulnerable and those most in need, banning direct-to-consumer pharmaceutical advertising, and ensuring merit-based appointments to agencies such as directors to the FDA, CDC, and NIH, aligning with executive branch health policies [160].

### 7.5. Administrative Overgrowth in U.S. Government Agencies and Universities

Over-employment of administrators in U.S. government healthcare agencies, universities, and colleges has become a significant concern for inefficiency and higher costs [186,187]. For example, from 1975 to 2005, the number of administrative personnel in higher education institutions—roles such as deans, legal staff, and compliance officers—increased by over 85% [188,189], outpacing both student enrollment and faculty hiring in universities, which makes no sense [186,190]. This expansion was driven partly by rising regulatory demands and increasing operational complexity, and to protect administrators themselves (i.e., hiring attorneys) has compromised the missions, efficiency, and optimal use of public and institutional resources [190].

Despite receiving substantial government grants and funding, many universities, medical schools in particular, have raised tuition fees at rates that outpace inflation and enrollment growth. The average tuition increase, estimated at 3.5% annually, is above inflation [188]. Some analysts suggest that this reflects a greater allocation of funds to administrative functions, support, and salaries, detracting from core academic missions like teaching, research, and student support [186,189]. On average, less than a third of university staff are involved in teaching [189]. Similar trends have been seen in government healthcare agencies, where the expansion of bureaucratic roles has significantly increased overhead costs [190] without improving health quality or outcomes.

In some institutions, administrative staffing levels, including legal and compliance teams—significantly outnumber core academic or clinical personnel. Diversity, equity, and inclusion (DEI) initiatives have further contributed to this excess and abuse of funds [187,190]. Disproportionate investments in administrative oversight [189], without corresponding support for teaching, research, academic excellence, or clinical care, have led to stagnation, inefficiency, or outputs, and substantial waste of funding [188].

### 7.6. Streamlining Workforce Inefficiencies in Healthcare and Higher Education

Given these dynamics, there is growing advocacy for reevaluating federal subsidies, financial aid, and loan programs, especially for wealthier universities and medical schools. The disproportionate rise in tuition costs in universities is increasing the burden on students, while campuses encourage students to take loans to pay for this [191]. Public funding mechanisms for well-endowed universities, including Ivy League institutions [188], should be reassessed to ensure alignment with national priorities such as affordability, innovation, access, fairness, and non-discrimination. However, meaningful reform in this area remains elusive [186].

The expansion of administrative roles with inflated salaries is a primary driver of rising tuition costs in both public and private universities, contributing to unsustainable expenditures that erode the quality of teaching and expected research outputs. The anticipated growth has not improved teaching quality, excellence, or hospital outcomes. Instead, spending in both sectors is diverted from direct services, like education and patient care, to sustain complex administrative infrastructures [186].

**Figure 3:**
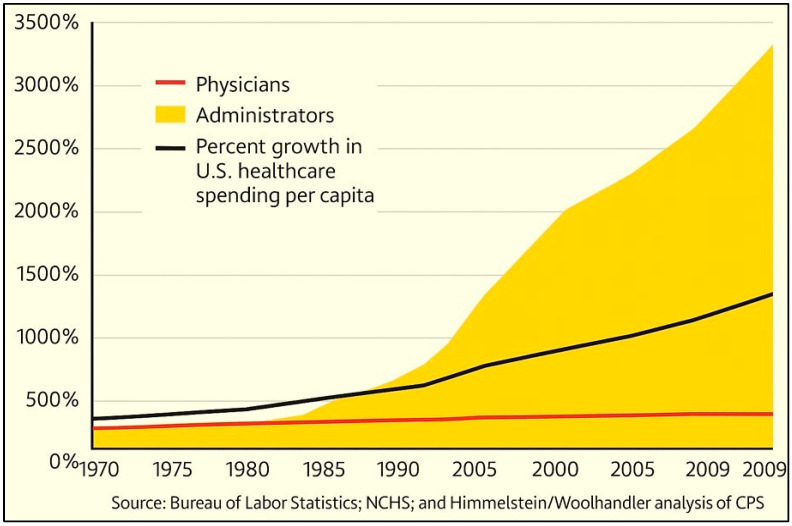
Illustrates an increase in U.S. healthcare spending per capita between 1970 and 2009. The figure also illustrates a 2300% increase in healthcare administrators compared to only a 10% increase in physicians during the same period (Source: Health Care Costs: A Primer, The Henry J. Kaiser Family Foundation): Bureau of Labor Statistics, the National Center for Health Statistics, and the United States Census Bureau’s Current Population Survey [187].

From 1970 to 2009, the number of healthcare administrators in the U.S. increased by over 2900%, while the number of practicing physicians grew by only 10% (Figure 3) [159]. This incongruence underscores the need for structural reforms to streamline operations and reallocate resources toward core functions, as carried out in the private sector. An estimated 40% reduction in managerial roles could significantly improve efficiency and accountability. Moreover, federal and state governments, universities, and institutions like the NIH should phase out unproductive academic programs that do not provide marketable skills or lead to well-paying jobs, as well as teaching and research programs that add no value to the nation.

Apart from administrative encouragement and the ease of taking out loans, the lack of skills training is one of the key reasons students are ill-equipped to repay said loans, thus getting trapped in debt after graduation [192,193]. Prioritizing frontline education [186] and healthcare services [187] would reduce unemployment, lower costs, and enhance quality and efficiency in these critical sectors.

### 7.7. Key Components Contributing to Escalating Healthcare Costs

Annual healthcare-related expenses represent a substantial financial burden not only for the government but also for the average American. Despite the promises made during the implementation of the ACA, insurance premiums continue to rise each year, along with the cost of prescription drugs, deductibles, and copayments—yet access to care remains limited. According to the Centers for Medicare & Medicaid Services (CMS), healthcare spending in the United States surged to USD 4.8 trillion in 2023 [194]. CMS projects that national health expenditure will reach USD 6.8 trillion by 2030 [195].

Compounding the problem is the widespread practice of defensive medicine—unnecessary tests and procedures conducted primarily to avoid litigation rather than to benefit patients—which contributes an estimated USD 46 to USD 300 billion annually to healthcare costs, accounting for roughly 3% of total national spending [196]. Figure 2 highlights three key cost drivers contributing to escalating U.S. healthcare expenses: (A) hospital services, (B) physician and clinical services, and (C) prescription drugs and medical devices [195].

**Figure 4:**
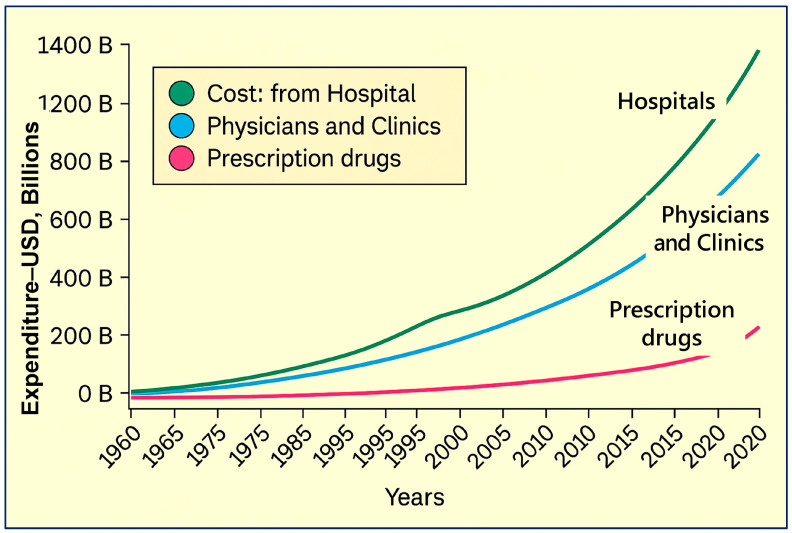
Computed annual key healthcare expenditures between 1960 and 2022 in the USA presented via three key line items: (A) hospitals, (B) physicians and clinics, and (C) prescription drugs and devices in escalating healthcare in America. Note that the inflection points coincided with the Affordable Care Act (ACA), which led to a major pharmaceutical price increase. Figure redrawn from National Health Expenditures 2022 Highlights (CMC.gov data).

To make things worse, because there is no universal cap, defensive medicine (unnecessary) expenses are added to healthcare costs, around USD 46 to USD 300 billion yearly, making up approximately 3% of national healthcare spending [196], with cumulative growth from 2008 to 2022—private insurance 62%, Medicare 41%, and Medicaid 22% (Source: KFF analysis of National Health Expenditure data). Figure 4 illustrates annual healthcare expenditures between 1960 and 2022 in the USA (obtained from multiple sources).

Meanwhile, spending in U.S. healthcare grew 7.5% in 2023, reaching USD 4.9 trillion (USD 14,570 per person)—health spending accounted for 17.6% of the nation’s Gross Domestic Product (CMS.gov data). The next section discusses the rationale for establishing the FNA.

## 8. Establishing the Food and Nutraceutical Agency

It is a once-in-a-lifetime opportunity for the US administration to restructure the FDA by separating its primary role in drug and medical device regulation from its oversight of food and nutraceuticals. The FDA should retain the approvals and regulations for drugs and medical devices and be redefined itself as the Drug and Medical Devices Agency (DDA). This would allow the new DDA to focus exclusively on pharmaceuticals and medical devices to improve transparency and efficiency in the approval process [157].

Congress should pass legislation to create a dedicated Food and Nutraceuticals Agency (FNA) to oversee all foods and over-the-counter nutraceuticals. Concurrently, this restructuring would provide a cost-effective, practical solution to overcome the failures and systemic challenges to restore public trust, and to enhance public health. The new legislation must also eliminate conflicts of interest in the new DDA and FNA agencies with the private sector businesses while addressing the FDA’s major deficiencies [157].

### 8.1. Rationale for Splitting the FDA

With a clear mandate, the FNA would ensure transparency, accountability, and efficiency while prioritizing public education to maintain a healthy population. Health priorities, shaped by profit-driven industries like health insurance and large hospital systems, often overshadow the critical importance of disease prevention [137]. Congress authorized the Dietary Supplement Health and Education Act for the FDA to regulate dietary supplements. However, the FDA and its Office of Dietary Supplement Programs [197] have failed to ensure transparency to expedite drug approvals [198], and to prevent unsafe food from entering the market. The dietary supplement industry, estimated to be worth $42 billion, continues to grow, with over 80,000 consumer products circulating [197].

The current inadequate food standards contribute to widespread illness, reduced productivity, and escalating healthcare costs. The FNA would implement stricter food standards, eliminate harmful additives and preservatives, enforce clear and honest food labeling, and promote access to healthy, functional foods, and orthomolecular nutrition/medicine, thereby improving public health [157]. These measures would reduce nutrient-deficiency-related disorders nationwide. Meanwhile, restructuring and redefining the FDA’s responsibilities would expedite the regulatory approval process, eliminate food deficiencies, and enhance food safety, benefiting the nation [198].

The FNA must be mandated to initiate an in-house research program to evaluate the presence and impact of aluminum, microplastics, glyphosate, and similar potentially harmful substances in food. In addition, it should determine and publish scientifically established safe maximum limits of these substances in food, expressed in parts per million (ppm) or mg/kg. Additionally, the FNA should release authoritative, research-based reports highlighting commonly consumed foods in the US that may contain toxic compounds, such as trans-fats and carcinogens. These publications would help inform the public, enabling individuals to make educated choices to avoid or moderate their intake of such harmful substances.

### 8.2. Expected Benefits from the FNA to the Government and the Public

Nutraceuticals are a broad category of foods and supplements. It includes dietary supplements, functional foods, and products with bioactive compounds that are scientifically confirmed [199]. Derived from natural sources, nutraceuticals provide health benefits beyond basic nutrition, focusing on children and vulnerable populations. These include dietary supplements, functional foods, and products enriched with bioactive compounds such as vitamins, minerals, and probiotics [199]. Creating the FNA would enhance transparency, accountability, and efficiency in regulation while empowering the public with better education for healthier choices. By addressing gaps in the FDA’s framework, the FNA would enable timely action, reduce waste and fraud, and prioritize public health—ultimately lowering healthcare costs for the federal government.

Functional foods have broader health benefits beyond well-known nutrients, providing sources of proteins, carbohydrates, vitamins, and dietary fiber [200]. They are enriched with many bioactive compounds, like polyphenols, alkaloids, and flavonoids. Functionally, they are reported to inhibit cell proliferation, engage in signal transduction, and induce apoptosis [200,201]. Nutraceuticals positively affect cardiovascular and immune health and are adjunct therapies to infection, including antioxidant, anti-inflammatory, and cancer properties [200]. Combining the orthomolecular approaches and holistic medicine can further improve the ’public’s benefits with less cost and adverse effects.

### 8.3. FNA Should Regain the Lost Credibility of Food and Nutraceutical Governance

There are several other key components to keep the public health. These include Omega-3 fatty acids that scavenge free radicals, which reduces the risk of chronic diseases like heart and respiratory disease and cancer [199]. Antioxidants within them, such as curcumin and sesame seed oil, inhibit inflammatory cytokines and, thus, cancer. Functional foods and nutraceuticals, therefore, are important parts of a healthy diet [105,201]. While dietary recommendations vary based on interest groups, personalized approaches are recommended to obtain the most benefits [200]. All these elements should come under the FNA.

To avoid industry bias, the FNA must initiate, preferably, an in-house research program to evaluate the presence and impact of aluminum [202], microplastics [203], and glyphosate, cookware that can potentially contaminate foods [204], and similar potentially harmful substances in food. In addition, it should determine and publish scientifically established safe maximum limits of these substances in food, expressed in parts per million (ppm) or mg/kg. Additionally, the FNA should release authoritative, research-based reports highlighting commonly consumed foods in the US that may contain toxic compounds, such as trans-fats and carcinogens. These publications would help inform the public, enabling individuals to make educated choices to avoid or moderate their intake of such harmful substances.

### 8.4. Benefits from the FNA to the Public

The FNA should establish stricter food standards, enhance labeling regulations, and promote access to healthy, functional foods, thereby improving public health and addressing deficiencies in the current FDA system [205]. Nutraceuticals, derived from natural sources, provide health benefits beyond basic nutrition, focusing on children and vulnerable populations. These include dietary supplements, functional foods, and products enriched with bioactive compounds, such as vitamins, minerals, and probiotics [157]. Establishing the FNA would ensure greater accountability and efficiency in regulating these products, enabling timely action and effectively addressing gaps in the existing FDA framework [157].

Structural reforms and the establishment of the FNA would also prioritize disease prevention, eliminating harmful contaminants and additives in food, minimizing the marketing process of food, making nutritious and safe foods available, and providing guidance to the public and the industry on changing lifestyles [3]. In addition, the empowerment of the FNA to regulate food, nutraceuticals, and OTC products would open doors for critically addressing the current public health challenges [157].

A straightforward mechanism for personalized dietary recommendations through the FNA would enable the public to make informed food choices to address undernutrition and obesity. This approach should consider the diminished nutritional value and heightened health risks linked to additives and excessive food processing, including products high in added fructose, corn syrup (e.g., bakery items), sugars in fruit juices and vegetable juices, and candy, and salt in chips. Promoting whole foods, minimally processed options, and balanced nutrient intake would enhance overall health and prevent diet-related diseases.

### 8.5. Challenges with the Proposed Legislative and Structural Changes

Challenges include legislative hurdles, resistance from groups with different interests and agendas, conflicts of interest, and potential short-term disruptions during the transition. Despite these challenges, separating the FDA into a Drug and Device Administration (DDA) and a separate Food and Nutraceutical Agency (FNA) would enhance efficiency, transparency, and accountability while potentially reducing costs. The proposed restructuring would streamline approval processes, eliminate bureaucratic overlaps, and improve oversight by dedicating each agency to distinguish regulatory responsibilities and accountability. These changes would lead to substantial cost reductions in healthcare, benefiting federal and state governments and, ultimately, the public. Maintaining rigorous safety and efficacy standards while ensuring that the DDA and FNA remain accountable to taxpayers would be crucial for trust and the success of this reform.

Pharmaceutical companies, medical institutions, and the public have markedly different perspectives and expectations on the FDA and its reform. Pharmaceutical companies likely resist changes introducing stricter oversight or disrupting established regulatory pathways, citing concerns about delayed drug approvals, increased costs, and diminished influence over FDA leadership. By contrast, the public generally supports reforms to enhance transparency, reduce conflicts of interest and costs, and ensure drug affordability and safety measures, which are crucial for restoring public trust in these agencies.

Medical institutions, while committed to patient care, may hold mixed views. A shift from the current sick-care model to an actual healthcare model would reduce the demand for hospitalizations and pharmaceuticals as the nation becomes healthier. Some institutions may advocate streamlined approval processes to accelerate access to innovative therapies, while others emphasize the need for rigorous oversight to ensure safety and efficacy. Balancing these competing priorities presents another challenge for policymakers considering FDA reform and its division into the DDA and FNA.

These debates and uncertainties will continue under the new US administration for some time as a new healthcare model and its structure evolves. However, structural reforms—including downsizing top-heavy, bloated administrative layers within healthcare agencies, are necessary to enhance efficiency, reduce redundancy, and improve accountability. While such changes may encounter resistance, they are crucial for establishing a more responsive and effective system.

Despite initial disruptions and protests, the proposed public health reforms and oversight mechanisms will yield clinical benefits and cost savings within 12 to 18 months by reducing chronic conditions like obesity, diabetes, Alzheimer’s disease, etc. These measures will alleviate the burden on hospitals and improve national health outcomes. Healthcare expenditure is expected to decline by one-third over the next few years, but achieving this will require political will, long-term vision, and collective commitment from policymakers, industry leaders, healthcare professionals, and the public.

## 9. Discussion

Preventive measures would not yield immediate results but will bring long-term savings, better and more effective care, including improved productivity, and maintain national health [205]. This requires political will, commitment, and long-term vision. However, prioritizing disease prevention will lead to conflicts with the short-term agendas of healthcare systems, pharmaceutical and insurance companies, some lawmakers, and economic interests. Congress must recognize these conflicts and counter lobbying influences for national benefit. Lobbying and financial incentives deter new policies and investment in preventive health, limiting sustainable public health improvements and cost savings. Forward-looking policies from the new US administration can overcome these barriers.

Primary, secondary, and tertiary prevention include clinical services such as nutritional education at all levels, counseling, and regular screenings to detect diseases early, enabling rapid interventions. Community-based approaches, like group sessions and faith-based institutions, are more cost-effective than hospital systems, promoting healthier lifestyles and reducing harmful exposures and injuries [5,137]. Workplaces, schools, and households can actively promote wellness by implementing AI-based health tools, supportive policies, and self-care initiatives to spread health messages and improve well-being effectively [7].

For example, maintaining vitamin D sufficiency improves most diseases and overall health, reducing absenteeism, boosting productivity, and increasing industry profitability [16,74]. Therefore, societies such as the American College of Occupational and Environmental Medicine (ACOEM) should take the lead in educating and guiding employers to keep employees healthy using such highly cost-effective means. Additionally, cybernetics, AI and machine learning (ML), and digital technologies must be established and integrated across all healthcare agencies to coordinate, modernize, and optimize system performance and identify failures and adverse effects of medications at the earliest possible stage [206].

The mentioned integration enables the application of cutting-edge science, real-time data acquisition and analysis, and rapid communication within and between agencies to quickly identify systemic inefficiencies, waste, fraud, and emerging issues [159]. Cybernetics, a transdisciplinary approach, employs feedback loops, recursions, and ML, processes vast amounts of input, and activates automated controls [206]. These systems facilitate the early detection of procedural violations, inefficiencies, and corruption, allowing for timely corrective actions [206,207].

Clear and straightforward food labeling, combating misleading marketing, and supporting legislation and accessible community counseling services to address negative health behaviors are essential for maintaining public health. Prioritizing nutrition, including macro- and micro-nutrients, daily physical activity, and sufficient sleep, can reduce non-communicable chronic diseases [110], improve quality of life, and pave the way for a healthier future. We can address this health crisis by collaborating with individuals, communities, and policymakers to build a resilient, thriving population.

With inevitable agency redundancies, excess funding for the proposed restructuring is unnecessary. Meanwhile, the author recommends a phased approach to reallocating healthcare budgets, starting with a 2% shift of the annual health budget toward public health and prevention initiatives over the next ten years, with an initial 5% reallocation in the first year to establish relevant programs [28]. Part of these funds would support establishing the FNA, to be situated away from the capital, in the country’s south. The FNA’s mandate would include comprehensive reforms in the food industry, such as stricter, unbiased regulations on additives, labeling, public education on nutrition, and marketing practices, which are key to improving national health. Strengthening food standards through public education campaigns, behavioral change initiatives, and legislative measures is crucial for long-term health improvements, especially for children and vulnerable populations.

With the incorporation of the above-mentioned technologies, the US healthcare system must shift from reactive to proactive, prioritizing disease prevention and enabling rapid, informed decisions based on real-time data [8]. The time to initiate this transformative change is now, as it represents the most effective strategy for achieving sustainable healthcare, improving the health of the population, and reducing healthcare costs for all.

The new U.S. administration and HHS must seize this critical moment to enact lasting healthcare reforms. While challenges are inevitable, they must not hinder bold, evidence-based policies that serve the public good. The proposed changes are crucial for lowering healthcare costs, reducing illness and absenteeism, and boosting national productivity. This article outlines foundational reforms, but further modernization and removing outdated, restrictive national guidelines and regulations are equally vital. Decisions made now will define the health and prosperity of future generations.

## 10. Conclusions

The information provided herein can be used as a White Paper to recognize health agencies and overall healthcare in the U.S. and elsewhere to make it more effective and less costly. The government must act swiftly to implement the policies outlined above to address the escalating healthcare crisis and costs. Preventable chronic diseases continue to drive the majority of healthcare expenses, straining both systems and budgets. Prioritizing prevention through public health initiatives can reduce hospital dependency, lower national healthcare expenditures, and significantly improve citizens’ quality of life. Delayed action will worsen the financial burden and further impair the nation’s health. At the same time, timely, prevention-focused reforms—such as establishing the FNA and reallocating funds—can lead to lasting savings and healthier communities [9].

Investing in the food and nutraceutical sectors, the FNA will address current major public health challenges, improve public health, improve the nation’s productivity, and stimulate economic growth by fostering innovation and creating new business opportunities. Bold government leadership in transforming healthcare policy will inspire similar action globally, reinforcing the U.S. as a model for health reform. All states in the US are expected to adopt and implement versions of these changes (e.g., prioritizing disease prevention) within their health system. With improved workforce health, reduced absenteeism, and increased national productivity, these efforts will enhance the country’s global competitiveness and set a new standard for proactive, sustainable healthcare governance [8]. The time to act is now.

## Data Availability

No new data were created or analyzed in this study. Data sharing is not applicable to this article.
